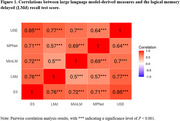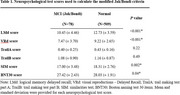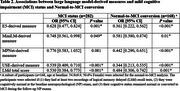# Developing New Digital Markers for Mild Cognitive Impairment: A Large Language Model‐Based Approach

**DOI:** 10.1002/alz70856_097820

**Published:** 2025-12-24

**Authors:** Zexu Li, Haochun Huang, Ting Fang Alvin Ang, Rhoda Au, Jinying Chen

**Affiliations:** ^1^ Dept of Anatomy & Neurobiology, Boston University Chobanian & Avedisian School of Medicine, Boston, MA, USA; ^2^ Bioinformatics Program, Faculty of Computing and Data Science, Boston University, Boston, MA, USA; ^3^ Department of Anatomy & Neurobiology, Boston University Chobanian & Avedisian School of Medicine, Boston, MA, USA; ^4^ Framingham Heart Study, Boston University Chobanian & Avedisian School of Medicine, Boston, MA, USA; ^5^ Slone Epidemiology Center, Boston University Chobanian & Avedisian School of Medicine, Boston, MA, USA; ^6^ Biomedical Genetics, Department of Medicine, Boston University Medical School, Boston, MA, USA; ^7^ Department of Epidemiology, Boston University School of Public Health, Boston, MA, USA; ^8^ Department of Neurology, Boston University Chobanian & Avedisian School of Medicine, Boston, MA, USA; ^9^ Department of Medicine/Section of Preventive Medicine and Epidemiology, Boston University Chobanian & Avedisian School of Medicine, Boston, MA, USA; ^10^ Data Science Core, Boston University Chobanian & Avedisian School of Medicine, Boston, MA, USA

## Abstract

**Background:**

The logical memory delayed (LMd) recall test has been widely used in screening tools for Alzheimer's disease (AD) and mild cognitive impairment (MCI). The test performance was evaluated by trained professionals based on the number of predefined key terms the participant accurately recalled from a previously narrated standard story. This process is subjective and labor‐intensive. We aim to develop novel, automated digital markers for MCI using large language models (LLMs) and transcriptions of recorded verbal responses from the LMd test.

**Method:**

We analyzed data from participants of the Framingham Heart Study who (1) had at least one audio transcription from the LMd test and (2) completed all the neuropsychological (NP) tests (in the same visit as (1)) used for assessing MCI based on the modified Jak/Bondi criteria. We developed automated measures that assessed participants’ LMd performance by using LLM‐derived text embeddings to estimate the semantic similarity between the standard LMd test story (72 words) and the transcriptions of verbal retellings (average 62 words). We implemented four measures using state‐of‐the‐art LLMs for short‐text embedding: E5, MiniLM, MPNet, and the Universal Sentence Encoder (USE). We evaluated these measures against the LMd score through two analyses. First, we used a generalized linear mixed model to assess the association between each measure and MCI status, adjusting for age, sex, and education. Second, we used logistic regression to assess the association between each measure at baseline and the conversion from cognitively normal to MCI within 15 years, adjusting for age, sex, education, and the conversion duration.

**Result:**

587 LMd recordings from 282 FHS participants (age at baseline: 53.7±9.8, 48.9% Female) were analyzed, with 78 recordings corresponding to MCI cases (Table 1). The LLM‐derived measures were correlated with the LMd score (Figure 1). Most LLM‐derived measures were negatively associated with MCI status (Table 2), with USE‐derived measure showing the strongest association (OR=0.539, *P* < 0.001). All LLM‐derived measures were negatively associated with normal‐to‐MCI conversion (Table 2), with USE‐derived measure being most sensitive (OR=0.344, *P* < 0.001).

**Conclusion:**

With further validation, these novel LLM‐derived measures can potentially serve as digital markers for MCI.